# Fabrication of Type-Variable Electronic Paper Using Electrophoretic Particle Loading with Multiple Bottom Electrode Structure

**DOI:** 10.3390/ma15062289

**Published:** 2022-03-20

**Authors:** Sang Il Lee, Dongjin Lee, Kunsik An

**Affiliations:** 1 Department of Mechatronics Engineering, Konkuk University, Chungju 27478, Korea; twosangone@gmail.com; 2 Department of Mechanical Design and Production Engineering, Konkuk University, 120 Neungdong-ro, Gwangjin-gu, Seoul 05029, Korea; djlee@konkuk.ac.kr

**Keywords:** electronic paper, E-paper, electrophoresis, cataphoresis, electrophoretic mobility, electrophoretic particle loading, type-variable display, nanoparticle transportation, electrokinetics

## Abstract

This study suggested the design of type-variable electronic paper with multiple bottom electrode structures and experimentally investigated the process mechanism of the electrophoretic particle loading method (EPLM) as an electronic ink injection method. The type-variable electronic paper was achieved by constructing the multi-electrode structure that had a structure of four electrodes that can independently apply voltage to one cell. By injecting electronic ink that mixes two types of particles with opposite charges into an electrically neutral color (blue) fluid, we realized electronic paper with a single color, and we then measured the optical characteristics of the panel. We used the EPLM to prevent charged particles that have lost their charge from being injected into the e-paper by using an electric field. In order to confirm the color expression and transmittance control effect using the multi-electrode structure, we conducted reflectance measurement and transmittance measurement experiments. Our experiments confirmed that the expression of more than five colors was possible and that the transmittance was controllable to a minimum of 13.50% and a maximum of 71.18%. This study provides an attractive method to create e-paper as a new form outside the framework of existing e-paper technology.

## 1. Introduction

Electronic paper (E-paper) is a new information display that combines the advantages of paper and digital display [1,2,3]. It does not require a backlight and has a wide viewing angle and memory that retains image information even when the power is turned off [4,5,6,7]. Recently, various products such as e-books, billboards, information boards, Electronic Shelf Labels (ESLs), and Points of Purchase (POPs) have been developed using e-paper due to its many advantages, such as easy readability [8,9,10]. While these advantages meet all the requirements of an ideal display, it is difficult to achieve them in a color display [11]. Thus, there has been a need for the development of additional technologies. In order to fulfill this need, several structural designs for color realization were proposed and demonstrated [12].

**Figure 1:**
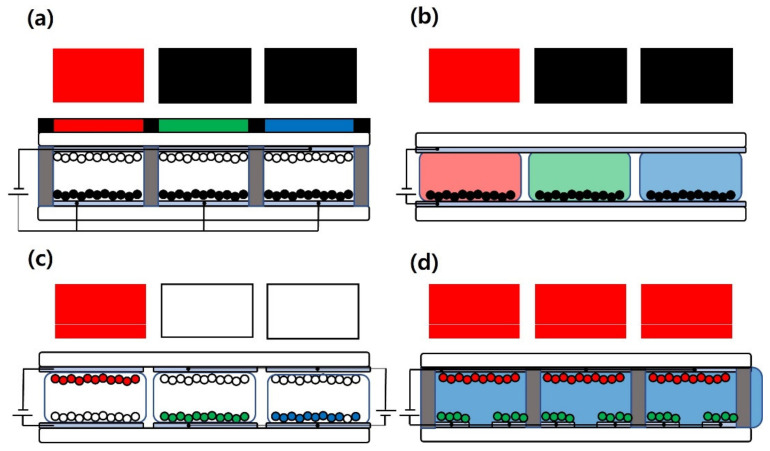
Various device structures of E-papers: (**a**) color filter structure, (**b**) color dye structure, (**c**) color particle structure, and (**d**) multi-electrode structure.

Figure 1 shows the color realization methods that have been reported in the last 10 years. The color filter approach (Figure 1a) enables some degree of color implementation by applying emissive display technologies, such as OLEDs [13,14] and LCDs [15,16]. However, the application of a sub-pixelation method for such a small pixel size and strong emissive light source has limitations in the realization of a perfect single-color implementation and high optical efficiency because of the use of color filters in electronic paper with relatively large cell size, over 100 μm, of the reflective type. As shown in Figure 1a, the other two sub-pixels for red implementation are black and white. The color dye method, as shown in the method in Figure 1b, was developed to display full color without color filter structures. It has many advantages, such as bright images, no color distortion, and a long lifetime. However, this method also has a limitation in the sub-pixelization method. E-paper is a reflective display that does not require a backlight. Therefore, the color filter or pixelation method causes a loss of reflected light, which is a fatal disadvantage that lowers the reflectance characteristics of electronic paper. In addition, if the size of the pixel is made larger, a vortex phenomenon occurs, and the behavior of the particles becomes unstable, so it is necessary to maintain a certain cell size. The method shown in Figure 1c was developed to overcome the optical loss associated with the color filter method and the disadvantages of the color dye structure method. In the color particle system (Figure 1c), three types of charged particles with different threshold voltages are injected into a cell, and the threshold voltage of the particles of each color are driven differently, as a pixelization system. This method can be used to implement a single color. However, the threshold voltage for electrophoresis of the nanoparticles is different from each other, and their driving voltages are also high, so the driving pulse is complicated. There are mainly two ways to inject nanoparticle inks: micro-bank type and capsule type. The micro bank-type electronic paper in which cells are divided using spacer is more complicated than that of the capsule type, which is produced by mixing a capsule with a binder. Likewise, micro-bank type electronic paper is known as a very high-level technology by filling the bank on the lower substrate with ink and laminating the upper substrate. In spite of these disadvantages, the development of the ink injection process to introduce the micro-bank type structure has been paid attention to because the micro-bank top electronic paper has optical properties better than the capsule type electronic paper such as haze, transmittance, and reflectance [17]. Currently, many methods such as inkjet printing and vacuum injection are being used, but many technical problems still remain. 

In this study, we suggested an electronic paper panel with multiple bottom electrode structures, as shown in Figure 1d. This panel consisted of three independent electrodes in each cell. Each cell of this display has one upper electrode on the upper substrate and two independently driven electrodes on the lower substrate. The suggested E-paper was filled with neutral fluid with the charged particles in each cell, which included three electrodes. The suggested E-paper had type variability that was used in both reflective type and transmissive type. The electro-optical characteristics of this display panel are determined by the ink injection methods and design of the cells, namely, the distance between the lower electrodes and upper electrode, the width of the electrode, the cell size, the cell height, and so on. 

## 2. Materials and Methods

**Figure 2:**
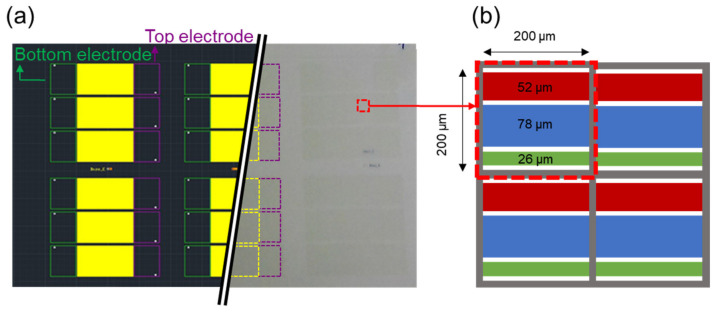
(**a**) The overall design, optical image, and (**b**) the cell design of E-paper with multiple electrode structures and its detailed information.

The multi-electrode type E-paper has a structure in which three electrodes are located in one cell-one common electrode on the upper substrate and three electrodes spaced on the lower substrate, as seen in Figure 2 in detail. The three electrodes at the bottom have different sizes of 26, 52, and 78 μm, respectively. The each of bottom electrodes was designed to have a different surface area, and the diverse cover area can be acquired by the combination of these three electrodes. By controlling the voltage on the electrodes, the aperture ratio can be controlled by arranging the nanoparticle ink. In addition, the thickness of the lower electrode is about 1 μm. The panel was made with a 30 mm × 30 mm board with a Lab scale, and the number of cells is 120 × 120. The three electrodes on the lower substrate were patterned and fabricated by photolithography. Various materials such as glass and film can be used for the lower substrate, but the glass substrate was used for the convenience of the experiment. The lower substrate was manufactured by applying a photolithography process, and the cell was formed by placing the partition pattern with SU-8 photoresist on the electrode pattern. The fabricated device has a cell size of 200 μm × 200 μm, a cell height of 23.4 μm, and an electrode width of 26.0 μm. 

The three electrodes and the spacer of the lower substrate were patterned and fabricated by a photolithography process using a photoresist SU-8. The multi-electrode electronic paper panel was completed by loading electronic ink on the lower substrate, aligning the upper and lower substrates, and packaging it using a bonder. The electronic ink used in this study was made by mixing white particles (first color) with a positively charged material (CCA) and a negatively charged material with black particles (second color) in an electrically neutral-colored fluid. The white particles were made of titanium, and the black particles were made of carbon. The particles were filled by coating them with polymethyl methacrylate (PMMA), and dispersant was also mixed to prevent the particles from agglomerating. The diameter, mass, and charge of charged particles were 450 nm, 6.6 μg, and 8 μC/g for white particles, and 250 nm, 3.3 μg, and −4 μC/g for black particles. The specific gravity of the particles was 3.2 g/m^3^. The reflectance was measured using a reflectance meter RT-200 (JNC Tech, Hwasung, Republic of Korea), and the transmittance was measured using a transmittance meter Spectrum paragon (PerkinElmer Co., Seoul, Republic of Korea). For the measurement method, the reflectance and transmittance of the color were measured by applying a driving voltage of ±2 V to the lower substrate electrode of the multi-electrode type electronic paper display panel. The reflectance of color was measured for the panel injected with ink mixed with white and black particles in a blue fluid, and transmittance was measured for the panel injected with ink mixed with black particles in a colorless fluid. In this case, the ink for transmittance control has a concentration of black particles of 3%, and the maximum transmittance and the minimum transmittance can be adjusted according to the particle concentration.

## 3. Results

### 3.1. Electrophoretic Particle Loading Method (EPLM)

The color expression of the E-paper was controlled by the charged particles in the electronic ink, but the unintended agglomeration of particles could hinder the clear expression of the color. Several reasons were suggested that cause the agglomeration during the manufacturing process. First, since positive or negative charges are accumulated on the surface of nanoparticles by electrostatic Coulomb force, charged particles become unstable. Secondly, when the nanoparticles have high surface energy by absorbing the energy during the fabrication process, the particles are aggregated to lower the surface energy. Finally, when the size of the nanoparticles is below a certain level, the Van der Waals force between the charged particles becomes greater than the gravity of the charged particles, and mutual aggregation occurs. 

When the agglomerated particles are injected into the panel, they interfere with the movement of other charged particles, causing large-scale particle aggregation. Therefore, it is necessary to disperse the electronic ink to restore the aggregated particles to their original state. However, even though the restoration of the particles in ink is performed, the particles cannot be perfectly redispersed to their original state. In this case, since the threshold voltage of the electronic paper increases, the driving voltage of the entire panel increases, thereby increasing power consumption. When an electric field is directly applied to the E-paper panel, electric charges are charged to the white and black charged particles of the electronic paper, and these charged particles move to the upper and lower portions of the electronic paper according to the electric field. At this time, some particles vastly different from the anticipated average charge become out of control because of several factors, such as the resistance of the fluid. Charged particles that do not reach the opposite electrode float unstably in the fluid inside the cell. These charged particles collide with other charged particles in motion and interfere with the normal movement of charged particles, which finally reduces the reflectance and response time of the panel. It was already reported that the injection of the charged particles outside the defined range into the cell-induced particle agglomeration and the lifetime of the panel was shortened [18]. In order to prevent the panel from these performances decline, it is necessary to screen particles within the acceptable range of charge during the injection process.

**Figure 3:**
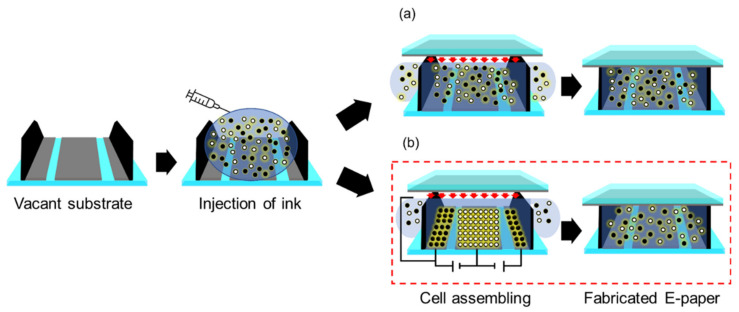
Fabrication procedures for loading methods of the E-ink with (**a**) conventional method and (**b**) electrophoretic particle load method.

Conventionally, the loading of the electronic ink between the lower substrate and the upper substrate was performed by simply injecting electronic ink into the existing bulkhead E-paper panel. A previous study confirmed that the optical properties of the E-paper display deteriorated when the concentrations of the E-ink injected into each cell were different from each other [19]. In this study, an electrophoretic particle loading method (EPLM) was used, in which an electric field was applied during the injection of E-ink to keep its concentration uniformly. The consistent maintenance of the ink during the fabrication process secures the optical properties of the display panel [20]. Figure 3 shows the schematic illustration for EPLM. The EPLM is a method of injecting electronic ink using the feature of three electrodes being formed on the lower substrate of a three-electrode type electronic paper display. After injection of the ink, the only particles in an acceptable range of charge were stuck to the bottom electrode by electrophoresis by applying a positive and negative voltage at the bottom electrode. Then, the upper substrate covered the bottom substrate by applying a driving voltage at the lower electrode and grounded voltage at the upper electrode. The overcharged and non-charged particles were filtered out with overloaded ink to the outside of the cell. Resultingly only the particles in the acceptable range of charge remained in the cell [21].

**Figure 4:**
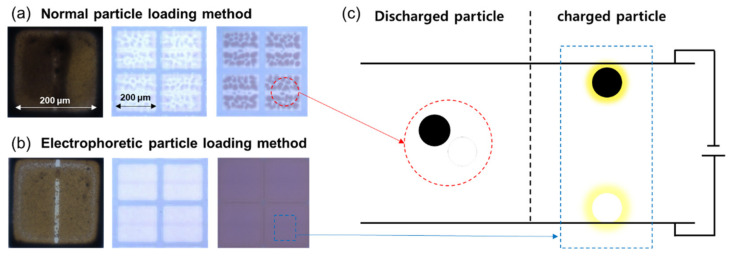
Optical microscopy images for E-paper fabricated by (**a**) conventional particle loading method and (**b**) electrophoretic particle loading method. (**c**) Schematic illustration for comparison of discharged and charged particles in the cell.

Figure 4 shows the optical microscopy images of the panels that were fabricated with and without EPLM. The panel shown in Figure 4a was impossible to adopt EPLM because the device consisted of only one electrode on the lower substrate. On the other hand, the panel shown in Figure 4b consisted of multiple electrode on the bottom substrate that was appropriate to adopt EPLM. The electronic ink was loaded into the cell under the voltage application of positive, negative voltage on the lower electrode and grounded voltage on the upper electrode. When the upper substrate was grounded, and the particles were aligned with the lower substrate, white and black particles were stuck to the lower electrode, and the particles that had lost the electric charge naturally flowed out with the overloaded fluid.

### 3.2. Demonstration of the Type-Variable Electronic Paper

**Figure 5:**
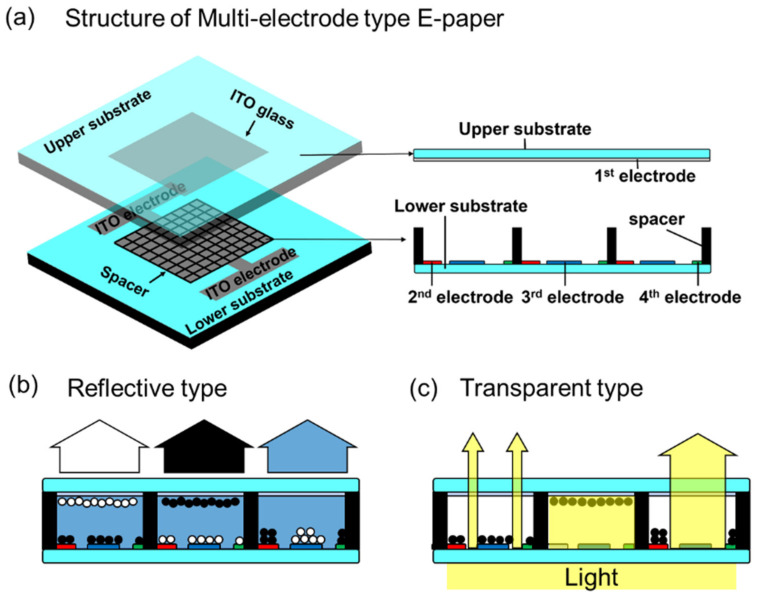
(**a**) Structure and driving method of multi-electrode type electronic paper and its schematic illustration for mechanism of (**b**) reflective type and (**c**) transparent type.

The multi-electrode type electronic paper has a structure in which one common electrode is placed on an upper substrate, and three electrodes are spaced apart from each other on a lower substrate in a unit cell. Figure 5a shows the three-dimensional structure of the proposed panel, in which a glass substrate coated with an ITO electrode was placed on an upper substrate. These structures are applicable for both reflective and transparent types, such as shown in Figure 5b,c. In these structures, the color of the reflective type could be expressed by the color of particles that stick to the upper electrode. Meanwhile, the color for the transparent type could be expressed by the areal density of the particles on the bottom electrode. The areal density was determined by the deployment of the particles that varies from the positive–positive–positive (PPP) voltage on three of the bottom electrodes to negative–negative–negative (NNN) voltage.

**Figure 6:**
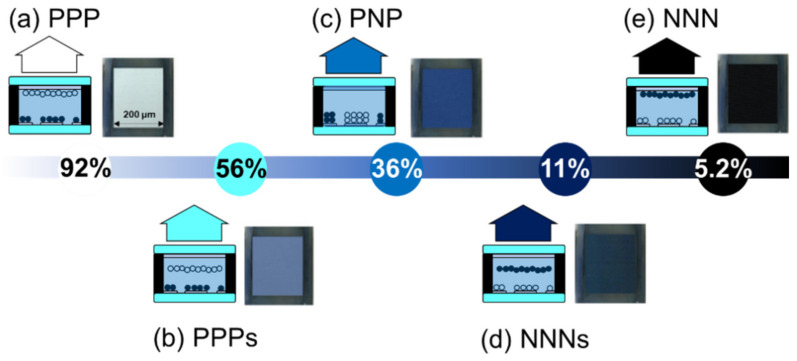
Schematic illustrations for the arrangement of the particles controlled by the voltage application and their reflectivity from 5.2% to 92% in reflective type E-paper. These experimental results included the reflectance of the glass.

The E-paper with a multi-electrode structure can express more than five colors. Figure 6a shows the appearance of a white film, in which positive voltage was applied to all three lower substrate electrodes (PPP), and black particles were placed on the lower substrate to express a single color of white on the upper substrate. The reflectance of white was 91.8%, which was the most similar to that of the white standard plate (97%). Considering the reflected light and absorbed light generated while passing through the upper substrate panel, we judged that almost perfect white was expressed. Figure 6b shows a color expression by combining the colors of white particles and blue fluid, and the color was expressed by shorting the upper substrate to separate the white particles from the upper substrate. The reflectance in this state was measured to be 55.8%. In addition, Figure 6c shows the blue color, applying positive voltage to the 1st and 3rd electrodes and negative voltage to the 2nd electrode (PNP) to place all the particles on the lower substrate, so that blue, the color of the fluid, is displayed on the upper substrate. The reflectance of blue was measured to be 36.3%, and in Figure 6d, a negative voltage was applied to the lower substrate (NNN), and the upper substrate was short-circuited. Additionally, as shown in Figure 6e, the reflectance of the state expressing black by grounding the upper substrate in the NNN state was 5.16%, showing the most similar reflectance to that of the black standard plate (1%). In this study, color was expressed using a combination of white, black, and blue due to the limitations of materials, but our experiments confirmed that color combination using RGB or CMY is also possible by selecting the particles properly.

**Figure 7:**
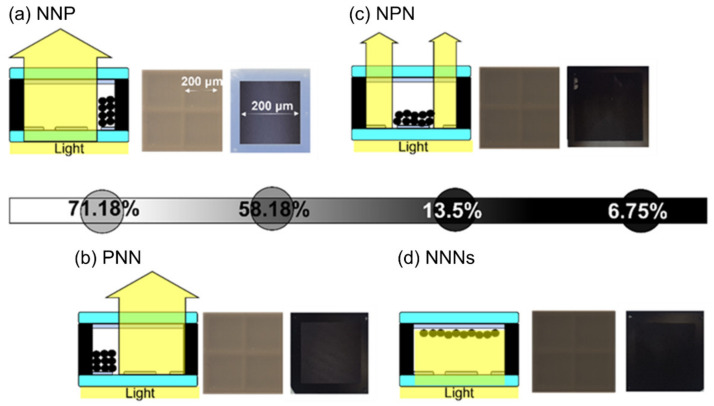
Schematic illustrations for the arrangement of the particles controlled by the voltage application and their transmittance from 6.8% to 71% in transmissive type E-paper with (**a**) NNP, (**b**) PNN, (**c**) NPN, and (**d**) NNNs bias.

**Figure 8:**
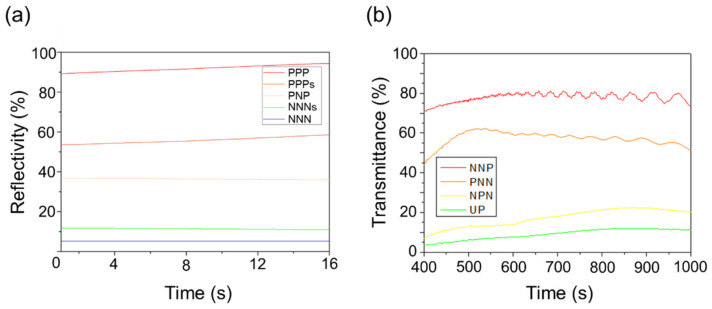
Optical properties of E-paper: (**a**) color reflectance; (**b**) transmittance measurement.

Figure 7 shows the state in which the transmittance is controlled by driving the sample. The left side of each figure is a schematic diagram, the center one is a microscopic observation, and the right one is visual observation. In Figure 7a, the transmittance is measured to be 71.18% with + voltage applied only to the 3rd electrode, which is the narrowest with an area of 20 μm (NNP), and the transmittance is enough to see the opposite side. Figure 7b shows that the sample is driven by applying a positive voltage only to electrode 1 with an area of 45 μm (PNN). Figure 7c shows a sample in which positive voltage was applied (NPN) only to electrode 2 (the largest of the three electrodes) with an area of 80 μm. Black particles were placed only in the middle of the lower substrate, and the upper substrate, compared with Figure 7d, was slightly transparent. Figure 7d confirms that the opposite side cannot be observed from the upper substrate because there were black particles on the upper substrate in a state where the negative voltage was applied to all three electrodes (NNN). This figure shows an example of the above-mentioned transmittance of 6.75%, which was at a level where the opposite side was hardly reflected. Another example exhibited 13.5% of the transmittance, and the opposite side was slightly reflected. The results showed that more varied transmittance control was possible by increasing the particle driving method and the number of lower electrodes. It resulted from the distribution of black particles located on the lower substrate. The aperture ratio of the film was determined by the distribution of black particles on the lower substrate, and the transmittance was determined by the aperture ratio. The area of each electrode was different by the bottom electrode designed with different sizes. Figure 8 shows a graph of the color reflectance and transmittance.

Table 1 shows the calculated aperture ratio of the film and the change in aperture ratio according to the voltage applied to the three electrodes. The rows named as (a) to (d) were the same as the arrangement of particles in Figure 7a–d. The aperture ratio and transmittance were not the same because the absorbed light and the reflected light were generated while light passed through the sample. Nevertheless, the results were consistent that the transmittance was increased by the aperture ratio. It confirmed that the aperture ratio varies according to the number or area of the electrodes, and the transmittance varies accordingly. In addition, it can be inferred that it is possible to control the transmittance in more ways by manufacturing the multi-electrode type electronic paper film by increasing the number or area of the electrodes. 

## 4. Discussion

The multi-electrode type E-paper display is a next-generation display technology that can realize a single color and control transmittance. In this study, to improve the process of injecting electronic ink into the existing electronic paper, we used the electrophoretic particle loading method (EPLM), which injects the electronic ink by applying an electric field, and we achieved color realization and transmittance control using the structure of the multi-electrode type electronic paper display. EPLM prevents the E-paper display panel from losing its particle charges out of the range, which eventually improves the lifespan. By using EPLM, type-variable E-paper was fabricated, whose structure consisted of multiple electrodes in one cell and three colors of electronic ink. The E-paper expressed single colors of white, black, and blue, and sky blue and dark blue expressed by combining voltage application on the electrodes. In addition, transmittance could be controlled from about 6% to 71% by controlling the aperture ratio of the panel injected with only the use of black particles. 

## 5. Conclusions

The type-variable E-paper can be used in both reflective and transmissive types that expand the usability of the device without any change in its structure. The suggested E-paper can be the platform as an initial point to be differentiated to diverse applications. Through this study, we discovered the possibility to overcome existing challenges in E-papers that will expand the demand for E-papers in the display industry. In the future, if the stability and reliability of the manufacturing process system are secured through additional research on the color and electrode structure of electronic ink, it is expected to be applied to various flexible electronic device fields. 

## Figures and Tables

**Table 1:** Aperture ratio of the fabricated E-paper under different voltage application on the lower electrodes.

	1st Electrode (45 μm)	2nd Electrode (80 μm)	3rd Electrode (20 μm)	Aperture Ratio
(a)	on	on	on	34.78
(b)	off	on	off	56.52
(c)	on	off	off	67.39
(d)	off	off	on	89.13
-	off	on	on	45.65
-	on	on	off	67.39
-	on	off	on	78.26
-	off	off	off	100

## Data Availability

Not applicable.
